# Effectiveness of Curcumin on Outcomes of Hospitalized COVID-19 Patients: A Systematic Review of Clinical Trials

**DOI:** 10.3390/nu14020256

**Published:** 2022-01-07

**Authors:** Amir Vahedian-Azimi, Mitra Abbasifard, Farshid Rahimi-Bashar, Paul C. Guest, Muhammed Majeed, Asadollah Mohammadi, Maciej Banach, Tannaz Jamialahmadi, Amirhossein Sahebkar

**Affiliations:** 1 Trauma Research Center, Nursing Faculty, Baqiyatallah University of Medical Sciences, Tehran 1435916471, Iran; amirvahedian63@gmail.com; 2 Immunology of Infectious Diseases Research Center, Research Institute of Basic Medical Sciences, Rafsanjan University of Medical Sciences, Rafsanjan 7718175911, Iran; 3 Department of Internal Medicine, Ali-Ibn Abi-Talib Hospital, School of Medicine, Rafsanjan University of Medical Sciences, Rafsanjan 7718175911, Iran; 4 Department of Anesthesiology and Critical Care, School of Medicine, Hamadan University of Medical Sciences, Hamadan 6515917495, Iran; fr_rahimibashar@yahoo.com; 5 Laboratory of Neuroproteomics, Department of Biochemistry and Tissue Biology, Institute of Biology, University of Campinas (UNICAMP), Campinas 13083-862, Brazil; paulcguest@yahoo.com; 6 Sabinsa Corporation, East Windsor, NJ 08520, USA; mmjd52@hotmail.com; 7 Cellular and Molecular Research Center, Research Institute for Health Development, Kurdistan University of Medical Sciences, Sanandaj 6617713446, Iran; asadollah.Mohammadi@muk.ac.ir; 8 Department of Hypertension, Chair of Nephrology and Hypertension, Medical University of Lodz, 93-338 Lodz, Poland; 9 Cardiovascular Research Centre, University of Zielona Gora, 65-046 Zielona Gora, Poland; 10 Department of Nutrition, Faculty of Medicine, Mashhad University of Medical Sciences, Mashhad 9177948564, Iran; jamiat931@gmail.com; 11 Applied Biomedical Research Center, Mashhad University of Medical Sciences, Mashhad 9177948564, Iran; 12 Biotechnology Research Center, Pharmaceutical Technology Institute, Mashhad University of Medical Sciences, Mashhad 91177948954, Iran; 13 School of Medicine, The University of Western Australia, Perth 6009, Australia; 14 Department of Biotechnology, School of Pharmacy, Mashhad University of Medical Sciences, Mashhad 9177948954, Iran

**Keywords:** COVID-19, SARS-CoV-2, symptoms, cytokine storm, curcumin, systematic review

## Abstract

Despite the ongoing vaccination efforts, there is still an urgent need for safe and effective treatments to help curb the debilitating effects of COVID-19 disease. This systematic review aimed to investigate the efficacy of supplemental curcumin treatment on clinical outcomes and inflammation-related biomarker profiles in COVID-19 patients. We searched PubMed, Scopus, Web of Science, EMBASE, ProQuest, and Ovid databases up to 30 June 2021 to find studies that assessed the effects of curcumin-related compounds in mild to severe COVID-19 patients. Six studies were identified which showed that curcumin supplementation led to a significant decrease in common symptoms, duration of hospitalization and deaths. In addition, all of these studies showed that the intervention led to amelioration of cytokine storm effects thought to be a driving force in severe COVID-19 cases. This was seen as a significant (*p* < 0.05) decrease in proinflammatory cytokines such as IL1β and IL6, with a concomitant significant (*p* < 0.05) increase in anti-inflammatory cytokines, including IL-10, IL-35 and TGF-α. Taken together, these findings suggested that curcumin exerts its beneficial effects through at least partial restoration of pro-inflammatory/anti-inflammatory balance. In conclusion, curcumin supplementation may offer an efficacious and safe option for improving COVID-19 disease outcomes. We highlight the point that future clinical studies of COVID-19 disease should employ larger cohorts of patients in different clinical settings with standardized preparations of curcumin-related compounds.

## 1. Introduction

The continuing COVID-19 pandemic caused by the SARS-CoV-2 virus has caused multiple waves of cases in most countries of the world and has now infected more than 3.4% of the global population [1]. Although more than 56% of the global population has received at least one dose of a COVID-19 vaccine, a large number of people remain unvaccinated, and this is not sufficient to stop the further spread of the virus [2]. The emergence of several SARS-CoV-2 variants with altered properties that impact virus characteristics such as transmissibility and virulence has further complicated efforts to end or control the pandemic. Moreover, some of these variants may even be capable of evading the protective effects of the vaccines [3]. Even asymptomatic individuals infected by SARS-CoV-2 can still spread the virus, and “long COVID” has become a concern as a cause of chronic debilitating illnesses [4].

The common symptoms of mild COVID-19 illness are predominantly fever, dry cough, and tiredness. In moderate to severe forms of the disease, patients may experience difficulty breathing, chest pains, and hypoxia, which may progress to acute respiratory distress syndrome (ARDS), total organ failure, and death [5]. Although the mechanisms underlying COVID-19 pathogenesis are still under investigation, altered immune responses in the host appear to play a critical role [5]. The infection and viral replication process can result in immune system activation and secretion of pro-inflammatory mediators referred to as a ‘cytokine storm’. In turn, this can cause acute inflammation, a hyper-immune response, coagulopathies, and thromboembolic sequelae, leading to damaging effects for the host [6,7].

Until the vaccines can be administered widely, the world still needs effective treatments to improve patient outcomes and reduce transmission of COVID-19 disease. This includes the use of antiviral drugs such as remdesivir, which disrupt viral replication [8], anti-inflammatory medications such as tocilizumab and dexamethasone to lessen cytokine storm effects [9], and anticoagulants such as heparin to decrease coagulopathy and thromboembolic complications [10]. In addition, several nutritional supplements and natural products with immunomodulatory properties have been tested as potential therapeutic adjuvants in the fight against COVID-19 [11].

The natural spice curcumin has received recent attention in treating diseases involving perturbations of the immune system and inflammation responses, such as COVID-19 [12,13,14,15]. Curcumin and other curcuminoids are the main bioactive ingredients of turmeric (Curcuma longa). They have been used for millennia in the traditional medicines of multiple cultures due to their anti-inflammatory, antioxidant, antibacterial, antiviral, antidiabetic, and neuroprotective properties [16,17,18,19,20,21,22,23,24,25]. Curcuminoids have received approval from the USA Food and Drug Administration (FDA), and these compounds have good tolerability and safety [26]. Furthermore, they have already been tested with some successes in clinical trials targeting various diseases [27,28,29,30].

Here, we have carried out a systematic review of clinical trials which assessed the effects of curcumin supplementation on various outcome measures, including symptoms, mortality, and inflammatory biomarker levels in COVID-19 patients with mild, moderate, and severe forms of the disease.

## 2. Methods

### 2.1. Setting and Search Strategy

The protocol of this study was carried out according to the Preferred Reporting Items for Systematic Reviews and Meta-analysis (PRISMA) statement [31]. We searched PubMed, Scopus, Web of Science, EMBASE, ProQuest, and Ovid databases up to 30 June 2021, to find all studies which examined the effects of curcumin and related compounds in the management of mild to severe COVID-19 in hospitalized patients. Additionally, the first 30 pages of Google Scholar (GS) were searched to find related articles. For the search strategy, the following MeSH terms and keywords were used: “novel coronavirus” OR “novel coronavirus 2019” OR “Wuhan coronavirus” OR “Wuhan pneumonia” OR “COVID-19” OR “SARS-CoV-2” AND “curcumin” OR “nano-curcumin” OR “curcuminoid” OR “curcuminoids”. Full details of the search strategy are given in Supplementary File S1. To further identify potentially related studies, the references of primary articles were also reviewed.

### 2.2. Eligibility Criteria

#### 2.2.1. Inclusion Criteria

Studies were eligible if they met all of the following inclusion criteria: (a) they were randomized or non-randomized controlled trials (RCTs), randomized crossover studies, or cluster-randomized trials; (b) the included patients with COVID-19, based on a positive—real-time reverse transcription PCR (real-time RT-PCR) analysis of respiratory tract samples, or imaging findings highly suspicious for COVID-19 (e.g., ground-glass pattern in chest X-ray); (c) the included patients with an age range of 18–85 years old; and (d) the patients had mild to severe disease based on national diagnosis and treatment guidelines [32].

#### 2.2.2. Exclusion Criteria

Studies were excluded if: (a) they enrolled participants who were pregnant or breastfeeding; (b) the patients had a history of hypersensitivity to turmeric or curcumin compounds; (c) the patients had a history of diseases such as severe renal failure (estimated glomerular filtration rate < 30 mL/min), hepatic failure (Child–Pugh score B or C), heart failure (ejection fraction < 40%), chronic lung disease, an active malignancy, an auto-immune disease, an immune system impairment such as human immunodeficiency virus, a gallbladder stone or active gastrointestinal bleeding; (d) the reports were conference articles, abstracts or protocols; and (e) the articles were published in non-English or non-Persian languages.

### 2.3. Study Selection

The selection of articles was made in three steps by two authors. First, the outputs of all six scientific databases were evaluated, and duplicate papers were removed. Two researchers screened the studies separately by title and abstract to identify seemingly related articles for a second screening. Following this, two researchers independently examined the complete texts of the remaining papers and identified studies that met the inclusion and exclusion criteria for the review. Disagreements between the two researchers were resolved by discussion. If disagreements persisted, a third author reviewed the study and made the final decision. To increase the quality of the review, a blind method was used with the journal and author names hidden.

### 2.4. Data Extraction

Information extracted from each study included the first author’s name, year of study, country, number of included patients in both intervention and control groups, age (mean), male (%), type of study, intervention, and main findings.

### 2.5. Quality Appraisal

The methodological quality of included manuscripts was assessed independently by two authors using the Jadad rating scale for RCTs [33]. This scale is a three-item, validated, and reliable scoring tool that focuses on randomization, blinding, and withdrawals/dropouts of the studies in question. Using this method, studies are given a total score of 0 to 5, with 5 being the highest and a score ≥3 considered high quality. Disagreements between the researchers were resolved as above.

## 3. Results

### 3.1. Search Outcomes

**Figure 1:**
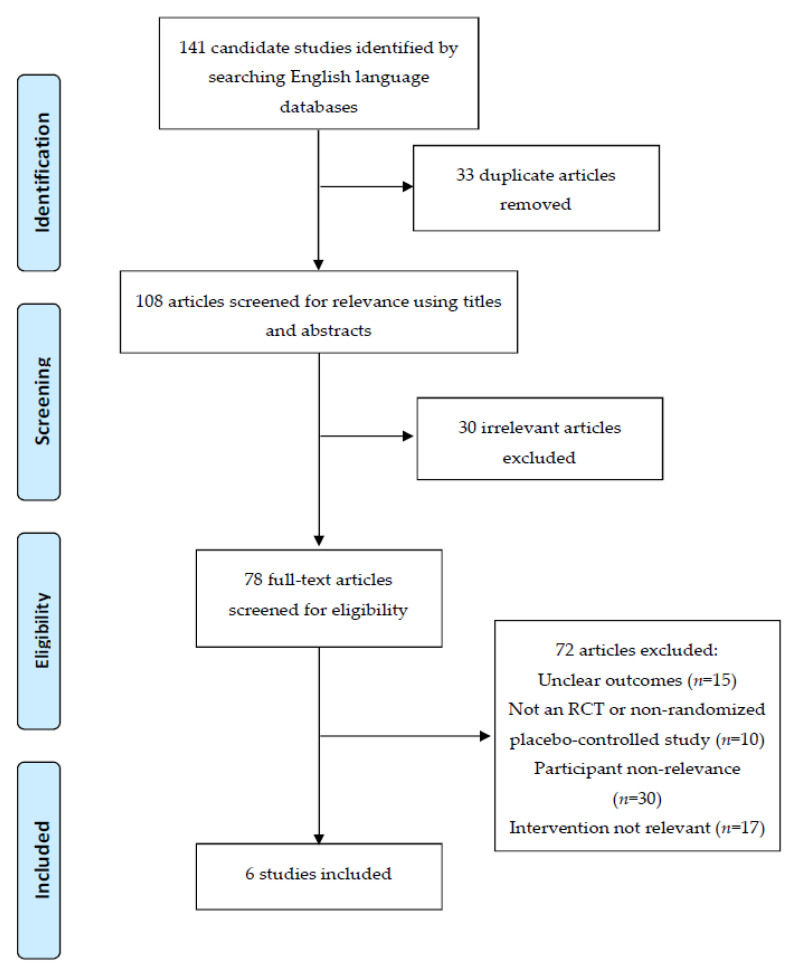
PRISMA (Preferred Reporting Items for Systematic Reviews and Meta-Analysis) flowchart detailing the disposition of screened, included, and excluded records.

The search strategy yielded a total of 141 candidate articles. After removing duplicate and irrelevant studies based on screening titles and abstracts, 78 articles remained. These were screened by full-text evaluation, and 72 records were excluded as they did not meet the criteria. The excluded articles either had unclear outcomes (*n* = 15), they were not RCT studies (*n* = 10), they did not include relevant participants (*n* = 30), or they did not use a relevant intervention (*n* = 17). Finally, six full-text articles were included in the study [34,35,36,37,38,39] (Figure 1). The characteristics of these studies are given in Table 1. The studies by Ahmadi et al. [34] and Saber-Moghaddam et al. [35] were from the Mashhad University of Medical Sciences. These used distinct study formats with different outputs from the same clinical trial (IRCT20200408046990N1). The study by Valizadeh et al. [36] and the two studies by Tahmasebi et al. [37,38] measured different endpoints from the same trial at the Tabriz University of Medical Sciences (IR.TBZMED.REC.1398.1314). The clinical study (CTRI/2020/05/025482) was carried out solely by Pawar et al. [39] in Maharashtra, India.

### 3.2. Quality Assessment

The five papers describing randomized, double-blind, placebo-controlled clinical studies [34,36,37,38,39] and the one non-randomized placebo-controlled study [35] were evaluated using the Jadad rating scale [33]. All of these studies had good quality (score ≥ 3), and three had the highest score of 5 [34,37,39]. Full details of the methodological quality assessment for included studies using the Jadad rating scale are given in Supplementary File S2.

### 3.3. Efficacy of Curcumin Therapy on the Clinical Manifestation of COVID-19

The resolution time for various symptoms related to COVID-19 infection was compared between the curcumin intervention and placebo control group across all six studies [34,35,36,37,38,39]. In all investigations, administration of oral curcumin therapy led to faster resolution of all symptoms related to COVID-19 than the control treatment. Reduced symptoms of cough (*p* = 0.043), chills (*p* = 0.013), myalgia (*p* = 0.043) and taste and smell disturbances (*p* = 0.032), and increased lymphocyte counts (*p* = 0.05) were significant in the intervention compared to the control group in the study by Ahmadi et al. [34] (Table 1 and Table 2). Similarly, Saber-Moghadam et al. [35] found a significantly reduced resolution time in the treatment group for symptoms of fever (*p* = 0.047), cough (*p* = 0.002), tachypnea (*p* = 0.031), chills (*p* = 0.004), and myalgia (*p* = 0.009), and significantly increased lymphocyte counts (*p* = 0.048) in the treatment group compared with the controls. Furthermore, oxygen saturation (SPO2) was significantly higher after 1 week (*p* = 0.022) and at discharge *p* < 0.001) in the treatment compared to the control group. Consistent with this, Alizadeh et al. showed that the nano-curcumin treatment led to significant reductions in most clinical manifestations including fever (*p* < 0.0001), cough (*p* < 0.0001) and shortness of breath (*P* < 0.0001) [36]. In the two studies by Tahmasebi et al. [37,38], the nanocurcumin treatment led to a significant (*p* < 0.05) improvement in fever, cough and dyspnea in the nanocurcumin-treated group compared to the placebo group (Table 1 and Table 2). In addition, Pawar et al. found significant (*P* < 0.05) reductions in dyspnea, pulmonary fibrosis and hospitalization duration in the curcumin/piperine treatment compared to the placebo group [39] (Table 1 and Table 2). 

### 3.4. Efficacy of Curcumin Therapy on the Mortality Rate of COVID-19 Patients

Mortality was reported in 4 studies comprising a total of 214 participants. Similarly, Valizadeh et al. found that the mortality rate in the curcumin group was 4 out of 20 patients in the intervention group and 8 out of 20 patients in the placebo group [36]. In a study of mild patients by Tahmasebi et al., the death rate in the curcumin group was 0 out of 20 and that in the placebo group was one out of 20 [37]. The same study showed that, among severe patients, the mortality rate in the intervention group was one out of 20 patients and 5 out of 20 in the placebo group. The second study by Tahmasebi et al. [38] showed that the mortality rate in the mild and severe COVID-19 disease was significantly lower (*p* < 0.05) in the curcumin compared to the placebo group. Finally, the randomized, double-blind, placebo-controlled clinical trial study conducted by Pawar et al. showed no deaths in the mild and moderate intervention groups [39]. However, there was one death out of 30 patients in the mild control group and 5 deaths out of 25 patients in the moderate control group. In addition, there were two deaths out of 15 patients in the severe intervention group compared to 5 deaths out of 15 patients in the severe control group (Table 1 and Table 2).

### 3.5. Efficacy of Curcumin Therapy on mRNA Expression and Pro-Inflammatory Cytokine Secretion

The study by Valizadeh et al. [36] showed that, compared to healthy controls, mRNA expression and secretion of interleukin (IL)-1β, IL6, TNFα, and IL18 were significantly increased in COVID-19 patients compared to controls. Based on real-time PCR, the expression and secretion of IL6 (*p* < 0.0001) and IL1β (*p* < 0.0001) were significantly reduced in the intervention group compared to controls. However, the intervention had no significant effect on IL18 mRNA expression and TNFα concentrations. Additionally, evaluation by ELISA showed similar results with significant reductions in IL1β (*p* = 0.0004) and IL6 (*p* < 0.0001) between the intervention and control groups and no significant difference in IL18 or TNFα levels (Table 1 and Table 2).

### 3.6. Efficacy of Curcumin Therapy on Frequency of T Helper (Th) 17 Cells and mRNA Expression of Th17 Cell-Related Factors

The study by Tahmasebi et al. analyzed 40 patients with severe COVID-19 disease admitted to the ICU and 40 patients with mild COVID-19 illness [37]. They found significant differences in mRNA levels of Th17-mediated factors for both the mild (*p* = 0.0001) and severe (*p* < 0.0001) patients in the nanocurcumin compared to the placebo-treated group after the intervention. In addition, the RAR-related orphan receptor γt (*p* = 0.002), IL-21 (*p* = 0.020) and GM-CSF (*p* = 0.002) were significantly reduced in mild patients, with no significant changes in any of these factors in the severe group (Table 1 and Table 2).

### 3.7. Efficacy of Curcumin Therapy on Regulatory T (Treg) Cell Frequency and Gene Expression of Treg Transcription Factor Forkhead Box P3

The second clinical study carried out by Tahmasebi et al. evaluated the effects of curcumin on 80 COVID-19 patients with either severe (*n* = 40) or mild (*n* = 40) disease [38]. In the intervention and placebo groups, the frequency of Treg cells, gene expression of transcription factor Treg forkhead box P3 (FOXP3) and cytokines (IL-10, IL-35, and TGF-β), and serum cytokine levels were measured in serum samples of mild and severe infected patients, in pre-and post-treatment with Nanocurcumin and placebo using ELISA. In mild patients, the curcumin treatment led to significantly enhanced serum levels of IL-10 (*p* = 0.016), IL-35 (*p* = 0.011), and TGF-β (*p* = 0.0002) compared with pre-treatment conditions. There was no significant change in any of these factors in the placebo group. At the same time, no significant differences were observed in secretion levels of TGF-β or IL-35 (718.6 ± 473 vs. 526.6 ± 398, *p* > 0.05) in the nanocurcumin and placebo groups, after the treatment period. In severe cases, nanocurcumin treatment significantly elevated the serum levels of IL-10 (*p* = 0.0018), IL-35 (*p* = 0.0013), and TGF-β (*p* = 0.0036) in the post-treatment nanocurcumin group when compared with the pre-treatment conditions. Moreover, a marked increase in the levels of IL-10 (*p* = 0.0009) was detected in the nanocurcumin-treated group in comparison with the placebo-treated group, and no significant differences were found in the levels of IL-10, IL-35 (*p* = 0.581) and TGF-β (*p* = 0.151) in the placebo group post-treatment vs. pre-treatment. Moreover, no significant differences were found in serum levels of IL-35 or TGF-β between the nanocurcumin- and placebo-treated groups (Table 1 and Table 2).

None of the studies reported side effects of the curcumin treatments. Thus, this could not be analyzed.

## 4. Discussion

This is the first systematic review to demonstrate the efficacy of supplementary curcumin treatment for improving general SARS-CoV-2 infection symptoms and mortality outcomes in mild, moderate, and severe COVID-19 patient groups. The benefits included decreased resolution time of several common COVID-19 symptoms, including cough, chills, myalgia, tachypnea, anosmia, and amelioration of lymphocyte counts. Furthermore, curcumin treatment decreased the mRNA expression and secretion of some, but not all, pro-inflammatory cytokines involved in the cytokine storm effect [36].

Consistently, the study by Tahmasebi et al. showed alterations in other parameters related to the cytokine storm, including reduced Th17 cell numbers, Th17 cell-related factors, and Th17 cell-related cytokines in the curcumin intervention group in both mild and severe COVID-19 patients [37]. From another angle, the second study by Tahmasebi et al. showed that the curcumin treatment led to changes in anti-inflammatory factors, including increased frequency of suppressor Treg cells, as well as elevated levels of transcription factor FOXP3, IL10, IL35, and TGF-β, and increased secretion of anti-inflammatory cytokines [38]. This is important as previous studies have demonstrated decreased levels of these components of the anti-inflammatory pathway in COVID-19 disease [40,41,42,43]. Thus, the increased levels of these parameters following the curcumin treatment might indicate restoration of the balance between pro-inflammatory and anti-inflammatory pathways. For patients with mild to severe COVID-19 disease, the mortality rate of the intervention group was significantly lower than that of the control group.

SARS-CoV2 infections are characterized by an imbalance in the immune system with hyper-activation of Th1 and Th17 cells and increased production of pro-inflammatory cytokines [44,45]. These effects can lead to the cytokine storm, which has been linked to more severe COVID-19 disease outcomes and increased mortality [46,47,48,49]. Notably, the study by Valizadeh et al. showed that a nanocurcumin formulation effectively modulated the inflammatory cytokines in COVID-19 patients [36]. These results are in line with those of other studies which tested the effects of nanocurcumin on Th17 cell-mediated inflammation in other diseases marked by hyper-inflammation or autoimmunity responses, such as multiple sclerosis [50,51], rheumatoid arthritis [52,53], and Alzheimer’s disease [54,55,56]. There is also accumulating evidence that curcumin treatment can modulate the cytokine cascade and cytokine storm caused by hyper-production of inflammatory cytokines [57,58]. This property appears to occur through inhibition of nuclear factor (NF)-κB and mitogen-activated protein kinase (MAPK) signaling, which drive cytokine production [15,59,60].

On the other hand, the expression of anti-inflammatory Treg cells is driven by a multiplex of transcriptional factors, including FOXP3, GATA3, E4BP4, and MAF [61,62]. In the study by Tahmasebi et al., the authors showed increased expression of the FOXP3 transcription factor associated with increased production of anti-inflammatory cytokines, such as IL-10 [38]. Although the immune response is responsible for host defense against various pathogens such as viruses, an excessive response of the pro-inflammatory cascade can result in severe damage to the host.

Here, we have shown that curcumin treatment—as an adjunct therapy—helps restore the balance between the pro-inflammatory and anti-inflammatory pathways and, at the same time, reduces the persistence of common COVID-19 symptoms and decreases mortality. Together, these findings support curcumin formulations as adjunctive therapy to reduce the hyper-inflammatory effect in COVID-19 patients and improve patient outcomes. However, curcumin has not been used as a single-drug therapy in clinical application, mainly due to its pharmacokinetic limitations. In its native form, curcumin exhibits poor bioavailability with low or undetectable concentrations in blood and extra-intestinal tissues due to poor absorption, chemical instability, rapid metabolism, and rapid systemic elimination [63,64]. However, analogs of curcumin and formulations such as adjuvants, nanoparticles, liposomes, micelles, and phospholipid complexes have been used to improve its bioavailability [63].

Several limitations should be considered in the interpretation of these results. First, the number of studies was low, with only six identified papers derived from three different clinical trials. Second, different curcumin formulations were tested, which did not allow direct comparison across the studies. Five of the investigations used two different dose regimes of SinaCurcumin (40 mg capsule, four times daily, for two weeks [34,35,36]; and 80 mg capsule, two times daily, for three weeks [37,38]) and one used a curcumin (525 mg)-piperine (2.5 mg) combination in tablet form administered twice a day [39]. Furthermore, the differences in mortality between the placebo and curcumin supplementation groups warrant further trials and review on a study-by-study basis to identify other potential factors for the findings. However, adequately controlled studies that account for key demographic variables, disease severity levels, and dosage regimes would address this. Thus, further studies should address the most effective posology regarding minimizing potential side effects and accounting for the effect of demographic factors and disease severity. These studies should include standardized formulations and dose regimes to allow direct comparisons of the results.

## 5. Conclusions

This review identified six studies that showed that adjunct treatment with different formulations of curcumin led to reductions in typical symptoms, duration of hospitalization, and deaths in COVID-19 patients with different levels of disease severity. At the same time, the curcumin treatment led to the amelioration of cytokine storm manifestation by reducing pro-inflammatory factors and stimulating anti-inflammatory pathways. Thus, these findings suggest that curcumin treatment may alleviate COVID-19 symptoms by restoration of the pro-inflammatory and anti-inflammatory balance. Furthermore, the study by Pawar et al. showed that curcumin-piperine supplementation led to fewer thromboembolic episodes following recovery from COVID-19 infections [39]. Considering that dose-escalating studies have demonstrated the safety of curcumin administration for up to three months [65], we suggest that treatment with this natural compound as a supplement should be evaluated both during and after hospitalization and in the post-vaccination stages to reduce the risk of thromboembolic events. However, we highlight the point that future evidence-based clinical studies of COVID-19 disease should employ standardized and well-characterized preparations of curcumin-related compounds. In addition, further studies are required using larger patient cohorts in outpatient, in-hospital, and post-COVID-19 settings to determine if curcumin supplementation is an efficacious and safe option for improving COVID-19 disease outcomes.

## Figures and Tables

**Table 1:** Characteristics of the studies included.

Reference.	Type of Studies	Sample Size		Age (Mean)	Male (%)	Intervention *	Conclusion	Quality Assessment
		Intervention	Control					
Ahmadi, Iran, 2020 [34]	Randomized triple-blind placebo-controlled clinical trial [IRCT20200408046990N1] Treatment duration: 2 weeks Follow-up: 2 weeks after treatment	30 mild to moderate COVID-19 patients	27 mild to moderate COVID-19 patients	43.15 ± 11.58	58.3%	Sinacurcumin^®^ soft gel 40 mg - Intervention group received 2 soft gels after breakfast and 2 soft gels after dinner daily for 2 weeks. - Placebo soft gels were prepared by the same company, with the same appearance containing all ingredients of medicine soft gel except curcumin with same dosing (2 soft gels twice daily after meal)	Positive effect of curcumin therapy. All symptoms except sore throat resolved faster in the treatment group, and the difference was significant for chills, cough, and smell/taste disturbances. CRP serum level was lower in the treatment group at the end of two weeks, and lymphocyte counts were significantly higher in the intervention group	5
Saber-Moghaddam, Iran, 2020 [35]	Open-label non-randomized placebo-controlled clinical trial [IRCT20200408046990N1] Treatment duration: 2 weeks Follow-up: 2 weeks after treatment	21 mild to moderate COVID-19 patients	20 mild to moderate COVID-19 patients	55.9 ± 15.16	65.9%	Sinacurcumin^®^ soft gel 40 mg - The intervention group received 2 soft gels after breakfast and 2 soft gels after dinner daily for 2 weeks. - Control group received 2 soft gel placebos twice daily after meal	Positive effect of curcumin therapy. Symptoms resolved significantly faster in the intervention group. Duration of supplemental O_2_ use and hospitalization also shorter in the treatment group	3
Valizadeh, Iran, 2020 [36]	Randomized, double-blind, placebo-controlled clinical trial [IR.TBZMED.REC.1398.1314] Treatment duration: 2 weeks	20 COVID-19 patients	20 COVID-19 patients + 40 healthy subjects	51.5 ± 8.2	76.2%	Sinacurcumin^®^ soft gel 40 mg - Intervention group received 160 mg nano-curcumin in four 40 mg capsules daily for 14 days. - Control group received the placebo capsule+ Both groups received: Betaferon 300 μg subcutaneously every other day until 5 days, Bromhexine 8 mg tablets every 8 h, and Atorvastatin 40 mg daily.	Positive effect of curcumin therapy. Nano-curcumin, as an anti-inflammatory herbal-based agent, may be able to modulate the increased rate of inflammatory cytokines (especially IL-1β and IL-6 mRNA expression and cytokine secretion) in COVID-19 patients, which may cause improvement in clinical manifestation and overall recovery	3
Tahmasebi, Iran, 2020 [37]	Randomized, double-blind, placebo-controlled Clinical trial [IR.TBZMED.REC.1398.1314] Treatment duration: 3 weeks	40 mild and severe COVID-19 patients	40 mild and severe COVID-19 patients + 40 healthy subjects	54.2 ± 9.1	60%	SinaCurcumin^®^ (Exir Nano) 80 mg -Intervention group received an 80 mg capsule two times daily (every 12 h) for 21 days. - Placebo group received a placebo capsule two times daily (every 12 h) for 21 days	Curcumin reduced the frequency of Th17 cells and related inflammatory factors in both mild and severe COVID-19 patients. Hence, it could be considered as a potential modulatory compound in improving patient inflammation	5
Tahmasebi, Iran, 2021 [38]	Randomized, double-blind, placebo-controlled Clinical trial [IR.TBZMED.REC.1398.1314] Treatment duration: 3 weeks	40 mild and severe COVID-19 patients	40 mild and severe COVID-19 patients + 40 healthy subjects	54.2 ± 9.1	60%	SinaCurcumin^®^ (Exir Nano) 80 mg - Intervention group received an 80 mg capsule two times daily (every 12 h) for 21 days. - Placebo group received a placebo capsule two times daily (every 12 h) for 21 days	In both mild and severe COVID-19 patients, nano-curcumin upregulated frequency of Treg cells, expression levels of FoxP3, IL-10, IL-35, and TGF-β, as well as serum levels of cytokines in the treatment group	4
Pawar, India, 2020 [39]	Randomized double-blind placebo-controlled clinical trial [CTRI/2020/05/025482] Treatment duration: 2 weeks	70 mild to severe COVID-19 patients; Mild (*n* = 30) Moderate (*n* = 25), and severe (*n* = 15) -Mild: (SpO2 > 94%) -moderate: (SpO2, between 90–94%) -Severe: (SpO2 < 90%)	70 mild to severe COVID-19 patients; Mild (*n* = 30) Moderate (*n* = 25), and severe (*n* = 15)	Range (18–85)	70.7%	Curcumin administered with piperine - Intervention group received Curcumin (252 mg) Complex^®^ (SamiDirect, India) dietary with (2.5 mg) Bioperine^®^ (SamiDirect, India) twice a day for 14 days from the day of admission. - Control group received a dose of probiotics (Nutrolin B Plus, which contains lactic acid Bacillus and Vitamin B; Ciplamed) twice a day for 14 days.	Positive effect of curcumin therapy. Showed early symptomatic recovery and could substantially reduce the duration of hospitalization in patients with moderate to severe symptoms, and fewer deaths observed in the intervention group	5

* In all trials, patients received standard of care based on the national COVID-19 guidelines. Investigational interventions are listed in the table.

**Table 2:** The main clinical and laboratory findings in the included studies.

	Saber-Moghaddam et al. [35]			Valizadeh et al. [36]			Ahmadi et al. [34]			Tahmasebi et al. [37]			Tahmasebi et al. [38]			Pawar s et al. [39]		
	Curcumin	Placebo	*p*-Value	Curcumin	Placebo	*p*-Value	Curcumin	Placebo	*p*-Value	Curcumin	Placebo	*p*-Value	Curcumin	Placebo	*p*-Value	Curcumin	Placebo	*p*-Value
Fever (°C) <37	0.62 ± 0.74	1.15 ± 1.35	**0.047**	37.5%	66.7%	**<0.0001**	2.86 ± 1.65	3.6 ± 3.3	0.373	Mild (0) Severe (10%)	Mild (30%) Severe (39.6%)	**<0.05**	Mild (0) Severe (10%)	Mild (30%) Severe (39.6%)	**<0.05**	80.0%	60.0%	NS
Oxygen saturation level %	94.33 ± 4.01	74.28 ± 22.1	**0.001**	-	-	-	-	-	-	-	-	-	-	-	-	-	-	-
Myalgia, N (%)	1.9 ± 0.83	3.44 ± 1.33	**0.009**	-	-	-	3.08 ± 2.75	4.38 ± 3.01	**0.043**	-	-	-	-	-	-	20.0%	33.3%	NS
Cough, N (%)	1.62 ± 0.8	3.89 ± 1.54	**0.002**	12.5%	50%	**<0.0001**	4.84 ± 4.29	6.96 ± 3.87	**0.043**	Mild (5%) Severe (10%)	Mild (20%) Severe (47%)	**<0.05**	Mild (5%) Severe (10%)	Mild (20%) Severe (47%)	**<0.05**	73.3%	73.3%	NS
Chills, N (%)	1.14 ± 1.31	2.55 ± 1.57	**0.004**	-	-	-	1.93 ± 0.46	2.6 ± 0.99	**0.013**	-	-	-	-	-	-	-	-	NS
Dyspnea	1.14 ± 0.85	1.85 ± 1.39	**0.031**	6.25%	8.33%	**<0.0001**	8.37 ± 3.92	8.62 ± 2.88	0.887	Mild (1%) Severe (5%)	Mild (5.2%) Severe (15%)	**<0.05**	Mild (1%) Severe (5%)	Mild (5.2%) Severe (15%)	**<0.05**	40.0%	80.0%	**<0.05**
Smell and taste	1.62 ± 1.07	1.44 ± 1.59	0.769	-	-	-	3.56 ± 2.01	5.14 ± 3.37	**0.032**	-	-	-	-	-	-	-	-	-
Pulmonary fibrosis	-	-	-	-	-	-	-	-	-	-	-	-	-	-	-	13.3%	93.3%	**<0.05**
Hospitalization duration (day)	5.05 ± 1.36	9.15 ± 4.28	**<0.001**	-	-	-	-	-	-	-	-	-	-	-	-	80.0%	33.3%	**<0.05**
Lymphocyte count	18.76 ± 6.75	11.99 ± 6.01	**0.048**	35%	75%	**<0.0001**	5.440 ± 62.22	2.198 ± 948	**0.05**	Mild (45%)	Mild (52%)	**<0.05**	Mild (45%)	Mild (52%)	**<0.05**	-	-	-
Serum TNF-α (pg/mL)	-	-	-	* 0.94 ± 0.41	1.09 ± 0.24	**NS**	-	-	-	-	-	-	-	-	-	-	-	-
Serum IL-1β (pg/mL)	-	-	-	* 0.56 ± 0.31	1.16 ± 0.27	**<0.0001**	-	-	-	-	-	-	-	-	-	-	-	-
Serum IL-6 (pg/mL)	-	-	-	* 0.58 ± 0.25	1.15 ± 0.32	**<0.0001**	-	-	-	-	-	-	-	-	-	-	-	-
Serum IL-10 (pg/mL)	-	-	-	-	-	-	-	-	-	-	-	-	M: 46.1 ± 20.8 S: 38.2 ± 18.2	M:43 ± 16.6 S:19.21 ± 9.4	**0.0094** **0.0009**	-	-	-
Serum IL-17 (pg/mL)	-	-	-	-	-	-	-	-	-	M: 0.69 ± 0.21 S: 0.76 ± 0.11	M: 0.92 ± 0.1 S: 0.95 ± 0.1	**NS** **NS**	-	-	-	-	-	-
Serum IL-18 (pg/mL)	-	-	-	* 0.93 ± 0.35	1.07 ± 0.35	**NS**	-	-	-	-	-	-	-	-	-	-	-	-
Serum IL-21 (pg/mL)	-	-	-	-	-	-	-	-	-	M:0.54 ± 0.31 S: 0.87 ± 0.18	M: 1.01 ± 0.16 S: 0.94 ± 0.11	**0.02** **NS**	-	-	-	-	-	-
Serum IL-23 (pg/mL)	-	-	-	-	-	-	-	-	-	M: 0.79 ± 0.23 S: 0.82 ± 0.21	M: 0.91 ± 0.15 S: 0.97 ± 0.1	**NS** **NS**	-	-	-	-	-	-
Serum IL-35 (pg/mL)	-	-	-	-	-	-	-	-	-	-	-	-	M: 718.6 ± 473 S: 225.5 ± 118.3	M:526.6 ± 398 S: 182.9 ± 97.4	**>0.05** **>0.05**	-	-	-
Serum TGF-β (pg/mL)	-	-	-	-	-	-	-	-	-	-	-	-	M:64.8 ± 32.7 S: 87.7 ± 50.3	M:61.7 ± 27.3 S:66.9 ± 54.44	**>0.05** **>0.05**	-	-	-
Serum GM-CSF (pg/mL)	-	-	-	-	-	-	-	-	-	M: 0.45 ± 0.23 S: 0.77 ± 0.15	M: 0.98 ± 0.15 S: 0.98 ± 0.12	**0.02** **NS**	-	-	-	-	-	-
T-helper 17	-	-	-	-	-	-	-	-	-	M: 2.68 ± 1.04 S: 3.26 ± 1.11	M: 4.25 ± 1.54 S: 4.98 ± 1.53	**<0.001** **<0.001**	-	-	-	-	-	-
RORɣt	-	-	-	-	-	-	-	-	-	M: 0.67 ± 0.18 S: 0.87 ± 0.14	M: 1.18 ± 0.13 S: 1.03 ± 0.13	**0.002** **NS**	-	-	-	-	-	-
Mortality	-	-	-	4/20 (20%)	**8/20 (40%)**	**NR**	-	-	-	0/20 in mild 1/20 (5%) in severe	1/20 (5%) in mild 5/20 (25%) in severe	**<0.0001**	0/20 in mild 1/20 (5%) in severe	1/20 (5%) in mild 5/20 (25%) in severe	**<0.0001**	**0/30 in mild** **0/25 in moderate** **2/15 (13.4%) in severe**	**1/30 (3.4%) in mild** **5/25 (8%) in moderate** **5/15 (33.4%) in severe**	**NR**

NR: not reported, NS: not significant, * according to real-time PCR; Bold fonts denote significant *p*-values.

## Data Availability

Not applicable.

## Supplementary Materials

The following supporting information can be downloaded at: https://www.mdpi.com/article/10.3390/nu14020256/s1, Supplementary File S1. Details of search strategy; Supplementary File S2. Methodological quality assessment for RCTs included studies using the Jadad rating scale.
