# Supplementary material for: Effectiveness of Curcumin on Outcomes of Hospitalized COVID-19 Patients: A Systematic Review of Clinical Trials
Source: Nutrients. 2022 Jan 7;14(2):256. doi: 10.3390/nu14020256 (PMC8779570; doi:10.3390/nu14020256)
Supplement: Supplementary file 1 [file nutrients-14-00256-s001.zip › Supplementary File S1.pdf]

Supplementary file S1

Scopus: 34 (TITLE ("Novel coronavirus" OR "Novel coronavirus 2019" OR "2019 novel coronavirus" OR "2019 nCoV" OR "Wuhan coronavirus" OR "Wuhan pneumonia" OR covid-19 OR "2019-nCoV" OR "SARS-CoV-2" OR "coronavirus 2019" OR "2019-nCoV") AND TITLE (Nano-curcumin OR curcumin OR curcuminoid OR curcuminoids))

Pubmed: 39 ("Novel coronavirus"[Title] OR "Novel coronavirus 2019"[Title] OR "2019 novel coronavirus"[Title] OR "2019 nCoV"[Title] OR "Wuhan coronavirus"[Title] OR "Wuhan pneumonia"[Title] OR covid-19 OR "2019-nCoV"[Title] OR "SARS-CoV-2"[Title] OR "coronavirus 2019"[Title] OR "2019-nCoV"[Title]) AND (curcumin [Title] OR curcuminoid [Title] OR curcuminoids[Title])

Proquest: 8 ti("Novel coronavirus" OR "Novel coronavirus 2019" OR "2019 novel coronavirus" OR "2019 nCoV" OR "Wuhan coronavirus" OR "Wuhan pneumonia" OR covid-19 OR "2019-nCoV" OR "SARS-CoV-2" OR "coronavirus 2019" OR "2019-nCoV") AND ti(curcumin OR curcuminoid OR curcuminoids)

Wos

**TITLE:** ("Novel coronavirus") *OR* **TITLE:** ("Novel coronavirus 2019") *OR* **TITLE:** ("2019 novel coronavirus") *OR* **TITLE:** ("2019 nCoV") *OR* **TITLE:** ("Wuhan coronavirus") *OR* **TITLE:** ("Wuhan pneumonia") *OR* **TITLE:** (covid-19) *OR* **TITLE:** ("2019-nCoV") *OR* **TITLE:** ("SARS-CoV-2") *OR* **TITLE:** ("coronavirus 2019") *OR* **TOPIC:** ("2019-nCoV") *Indexes=SCI-EXPANDED, SSCI, A&HCI, CPCI-S, CPCI-SSH, BKCI-S, BKCI-SSH, ESCI, CCR-EXPANDED, IC Timespan=All years*	124943
**TITLE:** (curcumin) *OR* **TITLE:** (curcuminoid) *OR* **TITLE:** (curcuminoids) *Indexes=SCI-EXPANDED, SSCI, A&HCI, CPCI-S, CPCI-SSH, BKCI-S, BKCI-SSH, ESCI, CCR-EXPANDED, IC Timespan=All years*	16028
#2 AND #1 *Indexes=SCI-EXPANDED, SSCI, A&HCI, CPCI-S, CPCI-SSH, BKCI-S, BKCI-SSH, ESCI, CCR-EXPANDED, IC Timespan=All years*	25

Embase

'novel coronavirus':ti OR 'novel coronavirus 2019':ti OR '2019 novel coronavirus':ti OR '2019 ncov':ti OR 'wuhan coronavirus':ti OR 'wuhan pneumonia':ti OR 'covid 19':ti OR 'sars-cov-2':ti OR 'coronavirus 2019':ti OR '2019-ncov':ti	123566
curcuminoids:ti OR curcuminoid:ti OR curcumin:ti	14881
#2 AND #1	28

Ovid

("Novel coronavirus" or "Novel coronavirus 2019" or "2019 novel coronavirus" or "2019 nCoV" or "Wuhan coronavirus" or "Wuhan pneumonia" or covid-19 or "2019-nCoV" or "SARS-CoV-2" or "coronavirus 2019" or "2019-nCoV").ti.	31414
(curcumin or curcuminoid or curcuminoids).ti.	2563
1 and 2	7
