# Supplementary material for: Effectiveness of Curcumin on Outcomes of Hospitalized COVID-19 Patients: A Systematic Review of Clinical Trials
Source: Nutrients. 2022 Jan 7;14(2):256. doi: 10.3390/nu14020256 (PMC8779570; doi:10.3390/nu14020256)
Supplement: Supplementary file 1 [file nutrients-14-00256-s001.zip › Supplementary File S2.pdf]

Supplementary file S2

Table S1: Methodological quality assessment for RCTs included studies using the Jadad rating scale

First Author, Year, Ref	Randomization		Blinding		Withdrawals and dropouts	Total score
	Randomization is mentioned	The randomization was adequate?	Blinding is mentioned?	The blinding of methodology was correct?	Losses and exclusions were described?	
Ahmadi, 2020, (1)	1	1	1	1	1	5
Pawar, 2020, (2)	1	1	1	1	1	5
Saber-Moghadam, 2020, (3)	0	0	1	1	1	3
Valizadeh, 2020, (4)	1	0	1	0	1	3
Tahmasebi, 2020, (5)	1	1	1	1	1	5
Tahmasebi, 2020, (6)	1	1	1	0	1	4

1. Ahmadi R, Salari S, Sharifi MD, Reihani H, Rostamiani MB, Behmadi M, et al. Oral nano-curcumin formulation efficacy in the management of mild to moderate outpatient COVID-19: A randomized triple-blind placebo-controlled clinical trial. Food Science & Nutrition. 2021;n/a(n/a).
2. Pawar KS, Mastud RN, Pawar SK, Pawar SS, Bhoite RR, Bhoite RR, et al. Oral Curcumin With Piperine as Adjuvant Therapy for the Treatment of COVID-19: A Randomized Clinical Trial. Frontiers in pharmacology. 2021;12:669362.
3. Saber-Moghaddam N, Salari S, Hejazi S, Amini M, Taherzadeh Z, Eslami S, et al. Oral nano-curcumin formulation efficacy in management of mild to moderate hospitalized coronavirus disease-19 patients: An open label nonrandomized clinical trial. Phytotherapy research : PTR. 2021.
4. Valizadeh H, Abdolmohammadi-Vahid S, Danshina S, Ziya Gencer M, Ammari A, Sadeghi A, et al. Nano-curcumin therapy, a promising method in modulating inflammatory cytokines in COVID-19 patients. International immunopharmacology. 2020;89(Pt B):107088.
5. Tahmasebi S, El-Esawi MA, Mahmoud ZH, Timoshin A, Valizadeh H, Roshangar L, et al. Immunomodulatory effects of nanocurcumin on Th17 cell responses in mild and severe COVID-19 patients. Journal of Cellular Physiology. 2021;236(7):5325-38.
6. Tahmasebi S, Saeed BQ, Temirgalieva E, Yumashev AV, El-Esawi MA, Navashenaq JG, et al. Nanocurcumin improves Treg cell responses in patients with mild and severe SARS-CoV2. Life Sciences. 2021;276:119437.
